# A novel serine protease, Sep1, from *Bacillus firmus* DS-1 has nematicidal activity and degrades multiple intestinal-associated nematode proteins

**DOI:** 10.1038/srep25012

**Published:** 2016-04-27

**Authors:** Ce Geng, Xiangtao Nie, Zhichao Tang, Yuyang Zhang, Jian Lin, Ming Sun, Donghai Peng

**Affiliations:** 1 State Key Laboratory of Agricultural Microbiology, College of Life Science and Technology, Huazhong Agricultural University, Wuhan 430070, Hubei, People’s Republic of China

## Abstract

Plant-parasitic nematodes (PPNs) cause serious harm to agricultural production. *Bacillus firmus* shows excellent control of PPNs and has been produced as a commercial nematicide. However, its nematicidal factors and mechanisms are still unknown. In this study, we showed that *B. firmus* strain DS-1 has high toxicity against *Meloidogyne incognita* and soybean cyst nematode. We sequenced the whole genome of DS-1 and identified multiple potential virulence factors. We then focused on a peptidase S8 superfamily protein called Sep1 and demonstrated that it had toxicity against the nematodes *Caenorhabditis elegans* and *M. incognita*. The Sep1 protein exhibited serine protease activity and degraded the intestinal tissues of nematodes. Thus, the Sep1 protease of *B. firmus* is a novel biocontrol factor with activity against a root-knot nematode. We then used *C. elegans* as a model to elucidate the nematicidal mechanism of Sep1, and the results showed that Sep1 could degrade multiple intestinal and cuticle-associated proteins and destroyed host physical barriers. The knowledge gained in our study will lead to a better understanding of the mechanisms of *B. firmus* against PPNs and will aid in the development of novel bio-agents with increased efficacy for controlling PPNs.

Plant-parasitic nematodes (PPNs) cause severe losses in a broad range of plants and agricultural crops worldwide^1^,^2^,^3^. To date, over 4100 species of plant-parasitic nematodes have been described^3^,^4^,^5^. Among them, the root-knot nematodes (*Meloidogyne* spp.), found throughout the world in tropical, subtropical and warm-temperate areas, and the cyst nematodes (including *Heterodera* and *Globodera* spp.), which are distributed worldwide, are the most economically important PPNs^3^. The damage caused by PPNs has been estimated at more than $US 150 billion per year, with more than half caused by *Meloidogyne* spp. and cyst nematodes^1^,^6^. Most PPNs, including *Meloidogyne* spp. and cyst nematodes, are soil-borne root pathogens that impede normal plant uptake of nutrients and water. Currently, chemical nematicides are the primary method used to control PPNs. However, these chemical nematicides are toxic, have variable efficacy, and are costly to the environment and human health^7^. Crop rotation and resistant crop cultivars have also been used as complementary strategies for the control of PPNs; however, the effectiveness of these strategies is limited^1^,^3^. Therefore, environmentally friendly, effective and affordable alternatives for PPN control are urgently needed.

Biological control has shown promise as an economically and ecologically friendly approach to reduce pest damages, although it can be ineffective owing to the complex niche of organisms or resistance occurring through longtime use. To develop novel nematicides, more investigations of novel microorganisms in their environments are needed. PPNs usually inhabit the soil and are subject to infection by indigenous bacteria and fungi, which suggests the possibility of using microorganisms to control PPNs^3^. Indeed, in the soil ecosystem, there are specific microorganisms, such as nematophagous fungi and bacteria, that have sophisticated strategies for trapping, killing, and digesting PPNs, often targeting specific developmental stages of their life cycles^1^. In the past decades, many microorganisms have been extensively studied and have shown great potential for the biological control of nematodes^1^, including more than 700 species of nematophagous fungus^8^ and a wide variety of nematophagous bacteria^9^. The nematophagous fungi include diverse phylogenetic groups, such as *Ascomycota*, *Basidiomycota*, *Zygomycota*, and *Chytridiomycota*^1^,^8^. *Bacillus*, *Pseudomonas*, and *Pasteuria* represent the dominant populations of nematophagous bacteria in soil^1^,^9^, especially *Bacillus nematocida*, and their toxicity against PPNs suggests they will be ideal biocontrol agents^10^. Moreover, nematicidal cry genes such as cry5B and cry6A from *Bacillus thuringiensis* have been identified and applied to GM crops to control *Meloidogyne* spp^11^,^12^.

*Bacillus firmus* is an aerobic, gram-positive, spore-forming bacterium, which was first identified in 1933^13^. *B. firmus* strains can be isolated from diverse environments and are widely used for different applications, including removing heavy metals from waste water^14^, utilizing their various extracellular enzymes and cellular endonucleases^15^,^16^,^17^,^18^. Recently, *B. firmus* was described as an important nematophagous bacterium. Several studies have demonstrated that *B*. *firmus* effectively controls several different PPNs, such as the root-knot nematode *Meloidogyne* spp^19^,^20^,^21^, the soybean cyst nematode^22^, the stem nematode *Ditylenchus dipsaci*^20^, and *Radopholus similis* in banana^20^,^23^. For mechanisms, *B. firmus* possesses lethal or paralytic activity against J2 larvae and has effectors that inhibit PPN egg hatching^19^,^20^,^21^. Additionally, a *B*. *firmus* strain isolated from soil in Israel has been developed as biological nematicidal agent by the Agro-Green Company and registered under the trade name of BioNem-WP in Israel. This strain has been shown to be highly efficient at controlling PPNs in both greenhouse and nursery conditions^21^.

Biocontrol of PPNs in the complicated and changing soil environment is now regarded as unlikely to be achieved via one toxin or one nematophagous bacteria because of easily occurring resistance. To obtain a durable nematicide, more microorganisms with different killing mechanisms are needed. As natural enemies of PPNs, nematophagous microorganisms offer a promising approach to control nematodes. Some produce traps to capture and kill the worms, while others act as internal parasites and produce toxins and virulence factors to kill the nematodes from within. *B. firmus* showed excellent control of PPNs and has been produced as a commercial nematicide. However, its nematicidal factors and mechanisms are not yet clear. In this study, we isolated a strain of *B. firmus* DS-1 with a remarkable ability to kill *M. incognita* and soybean cyst nematode. To elucidate its nematicidal factors and mechanisms, we sequenced the complete genome of DS-1 and identified a novel nematicidal virulence factor, Sep1. We further showed that Sep1 could degrade multiple cuticle-associated protein and destroyed host physical barriers due to its serine protease activity. Our findings reveal the biocontrol factors and mechanisms of *B. firmus* against nematodes and uncover a different mechanism for possible applications in PPNs control.

## Results

### *B. firmus* DS-1 has high activity against *M. incognita* and soybean cyst nematode

**Figure 1:**
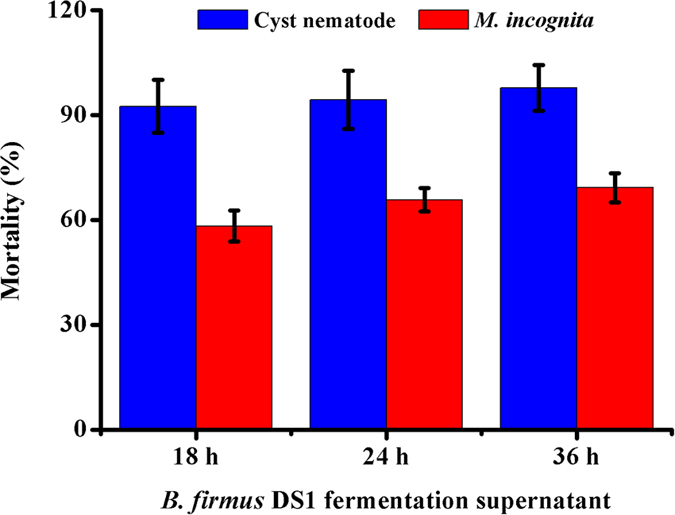
The bioassay of *B. firmus* DS-1 against *M. incognita* and soybean cyst nematode. The nematicidal activities of *B. firmus* DS-1 were tested using *M. incognita* and soybean cyst nematode as targets. Toxicity was assessed by calculating the average mortality rate of animals treated with *B. firmus* DS-1 fermentation supernatant taken at different time points. The mortality (20–30 for each sample) was calculated three days after treatment. The data are presented as the mean ± SD (n = 3).

*Bacillus firmus* DS-1 was isolated from marine sediment off the coast of the South China Sea^24^. To determine whether *B. firmus* strain DS-1 has nematicidal activity against PPNs, we cultured the *B. firmus* DS-1 for 18 h, 24 h and 36 h and then centrifuged the samples to collect the fermentation supernatant. Then, assays measuring the lethality of the fermentation supernatant against the PPNs *Meloidogyne incognita* and soybean cyst nematode were conducted. The results suggested that the fermentation supernatant of DS-1 from 18 h to 36 h was highly toxic to *M. incognita* and caused mortality rates of 59.5% to 70%, but there is no statistically significant difference between any two timepoints. The maximum activity appeared in the 36 h fermentation supernatant of DS-1 and caused 70% nematode mortality (Fig. 1). Similar results were also observed for the soybean cyst nematode. The mortality rate was 92.54% with the 18 h supernatant, while the 36 h fermentation supernatant of DS-1 showed a very high mortality rate of 97.81% (Fig. 1). These results demonstrated that *B. firmus* DS-1 has high activity against *M. incognita* and soybean cyst nematode and showed higher activity against soybean cyst nematode compared with *M. incognita*. These findings are consistent with previous reports that some *B. firmus* strains effectively control several different PPNs^19^,^20^,^21^.

### The genome of *B. firmus* DS-1 reveals a novel nematicidal virulence factor, Sep1

To identify the nematicidal virulence factors in *B. firmus* strain DS-1, we sequenced the genome of *B. firmus* DS-1 (accession number APVL00000000) with Illumina sequencing technology by BGI Shenzhen^24^. Using the reported nematicidal virulence-associated genes, including Bace16 of *Bacillus nematocida*^10^, Bmp1 from *Bacillus thuringiensis*^25^, and all other proteases mentioned in Bmp1 research^25^, as well as the hemolysin superfamily proteins, peptidase_S8, peptidase_M6, peptidase_M7, and peptidase_M48 superfamily proteins, a whole genome InterproScan analysis of the *B. firmus* DS-1 genome was conducted, and the results showed that the *B. firmus* DS-1 genome harbors multiple potential extracellular protease genes in the genome (Table 1).

To assess the nematicidal activity of these proteins, we cloned 13 genes into the expression vector pET-28a and transformed them into *E. coli* BL21 (DE3). The bioassay results showed that the recombinant protein EWG10090, expressed from *E. coli*, showed significant nematicidal activity against *C. elegans* N2, with 73.2% mortality. No other recombinant proteins showed significant nematicidal activity against *C. elegans* N2. Therefore, protein EWG10090 was selected for further study and designated Serine Protease 1 (Sep1) according to its similarity to annotated intracellular serine proteases. The recombinant proteins EWG13067 and EWG10233 also showed nematicidal activity against *C. elegans* N2, with 30% and 35% mortality, respectively.

**Figure 2:**
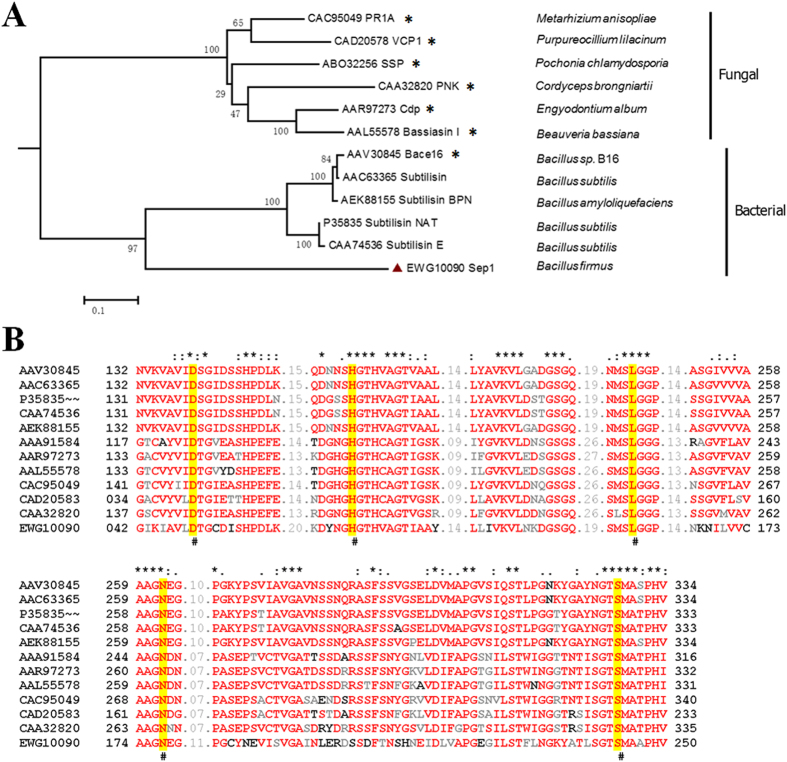
The phylogenetic tree (**A**) and multiple sequence alignment (**B**) of Sep1 with other selected nematicidal proteases. The species and GenBank accession numbers of other selected nematicidal proteases are shown. * Indicates the 7 reported proteases that have nematicidal activity. The amino acid sequences were aligned with the ClustalW program, and a neighbor-joining tree was generated. The final phylogenetic tree was constructed with MEGA4 software. The bar at the bottom of the figure represents the number of amino acid substitutions per site. The 5 conserved catalytic triads for serine protease activities are indicated by #.

The bioinformatics analysis showed that the Sep1 protein has 321 amino acids and contains a typical peptidase S8 superfamily conserved domain, which is found in the subtilase family of serine proteases. It has been reported that cuticle-degrading proteases produced by several nematicidal fungi belong to the peptidase S8 superfamily^26^,^27^,^28^. To better understand the function and evolution of this protein, we compared the Sep1 sequence with 11 reported S8 superfamily proteins from several fungi and bacteria species, including 7 reported nematicidal cuticle-degrading proteases. The phylogenetic tree of the proteases is shown in Fig. 2A. The serine proteases from bacteria and fungi are separated into two branches very clearly, which reflects their different sources. However, Sep1 is far from the fungal serine protease clade, while its bootstrap value of 97 indicates that Sep1 is close to the bacterial serine protease clade but belongs to another subclade, suggesting that Sep1 is different from the known bacterial serine proteases. Additionally, the sequence alignment of the available serine proteases showed that *B. firmus* Sep1 has a low amino acid sequence identity to all the reported bacterial serine proteases, with a 36–48% identity (Figure S1). This indicates that Sep1 is variable among these proteins, except for several residues near the 5 conserved catalytic triads that are probably essential for serine protease activity and are conserved in all the proteases, including Sep1 (Fig. 2B). The low sequence identity and distant evolutionary relationship with known serine proteases indicate Sep1 is a novel bacterial serine protease.

### Sep1 shows high toxicity against *C. elegans* and *M. incognita*

**Figure 3:**
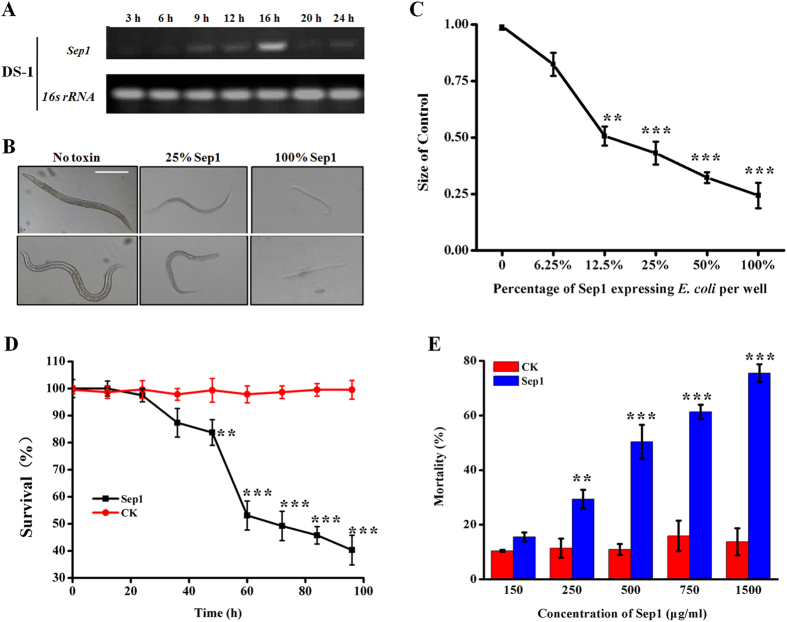
Sep1 protein shows toxicity against nematodes. (**A**) The transcript levels of *sep1* mRNA in *Bacillus firmus* DS-1 at different phases of bacterial growth. (**B**) Growth assay of Sep1 against *C. elegans*. All nematodes are shown at the same magnification (scan bar 100 μm). (**C**) The growth inhibition curve for the growth assay. The highest dose of (100%) represents Sep1-expressing *E. coli* BL21 (OD_600_ = 0.6); other doses were diluted with *E. coli* BL21 harboring an empty vector (Control). The inhibition percentage was the ratio of average worm size treated with Sep1 relative to the average size of control animals. (**D**) The mortality assays of Sep1 against *C. elegans*. The lethal activity of *E. coli*-expressed Sep1 (OD_600_ = 0.6) was tested with N2 L4 nematodes at different times. The same amount of *E. coli* BL21 harboring an empty vector was used as a control (CK). Each point represents the average result from 20–30 animals. (**E**) Mortality assay of Sep1 proteins against *M. incognita* J2. The 1 μg/ml RES solution-treated animals were used as controls (CK). Each point represents the average result from 50–60 animals. All the data are shown as the mean ± SD (n = 3), statistical comparisons between the values of detect samples and CKs were performed using a paired *t*-test, and significant differences were determined according to a threshold of *p < 0.05; **p < 0.01, and ***p < 0.001.

Virulence factors should be expressed during bacterial growth. Therefore, we analyzed the transcript levels of *sep1* mRNA in *B. firmus* DS-1 at different growth phases by RT-PCR. The results showed that *sep1* was expressed from 9 to 24 h, while transcript levels were relatively higher during the exponential phase of growth (12–16 h) (Fig. 3A). To determine if Sep1 contributes to the nematicidal activity of *B. firmus* DS-1, we wanted to construct a *sep1* deletion mutant strain of DS-1. However, we could not obtain this mutant because the constructed vector for *sep1* gene knockout could not be transformed into this strain. Therefore, we assessed the Sep1 activity against *C. elegans* by expression in *E. coli* and *M. incognita* by purified Sep1 protein only.

We first evaluated the nematicidal activities of Sep1 against a free-living nematode, *C. elegans,* using a growth assay and lethality assay. For the growth assay, the nematicidal activities of Sep1 expressed in *E. coli* BL21 were tested using L1 animals of *C. elegans* wild-type N2 as targets under microscopic observation. The *E. coli* BL21 harboring the empty vector pET28a was used as a negative control. The results showed that control nematodes were healthy and vigorous. However, the nematodes cultured with Sep1-expressed *E. coli* were significantly smaller than those cultured with control *E. coli* BL21. Even at a dose of 25% Sep1-expressed *E. coli*, the challenged nematodes showed a significantly smaller size compared with the control (Fig. 3B). We further quantified the results of the above growth assay using ImageJ as previously described^29^. These results showed that the inhibition percentage was increased with the increased gradient of Sep1-expressed *E. coli* content, indicating Sep1 has a significant growth inhibitory effect on *C. elegans* N2 L1 animals (Fig. 3C). We also tested the lethal activity of the expressed Sep1 protein in *E. coli* against *C. elegans* N2 L4 animals. The results showed that Sep1 caused significant lethality in *C. elegans* whereas no significant lethal activity was observed for *E. coli* BL21 harboring an empty vector (pET-28a/BL21, negative control) (Fig. 3C).

To evaluate the nematicidal activities of Sep1 against PPNs, different doses of *E. coli*-purified Sep1 proteins (Figure S2) were used. The *M. incognita* J2 animals (40–50 for each dose) were used as targets. The results showed that Sep1 caused significant lethality in the *M. incognita* J2 animals. With the increase in Sep1 concentration, the nematode mortality increased gradually. For example, when the Sep1 concentration was 150 μg/ml, it produced 18.65% mortality, while when the Sep1 concentration was 500 μg/ml, the mortality of *M. incognita* J2 animals reached 50.36% (Fig. 3C). Thus, the above results indicate that the Sep1 protein from *B. firmus* DS-1 is a virulence factor that has toxicity against the free-living nematode *C. elegans* and the PPN *M. incognita*.

### Sep1 protein exhibit serine protease activity

**Figure 4:**
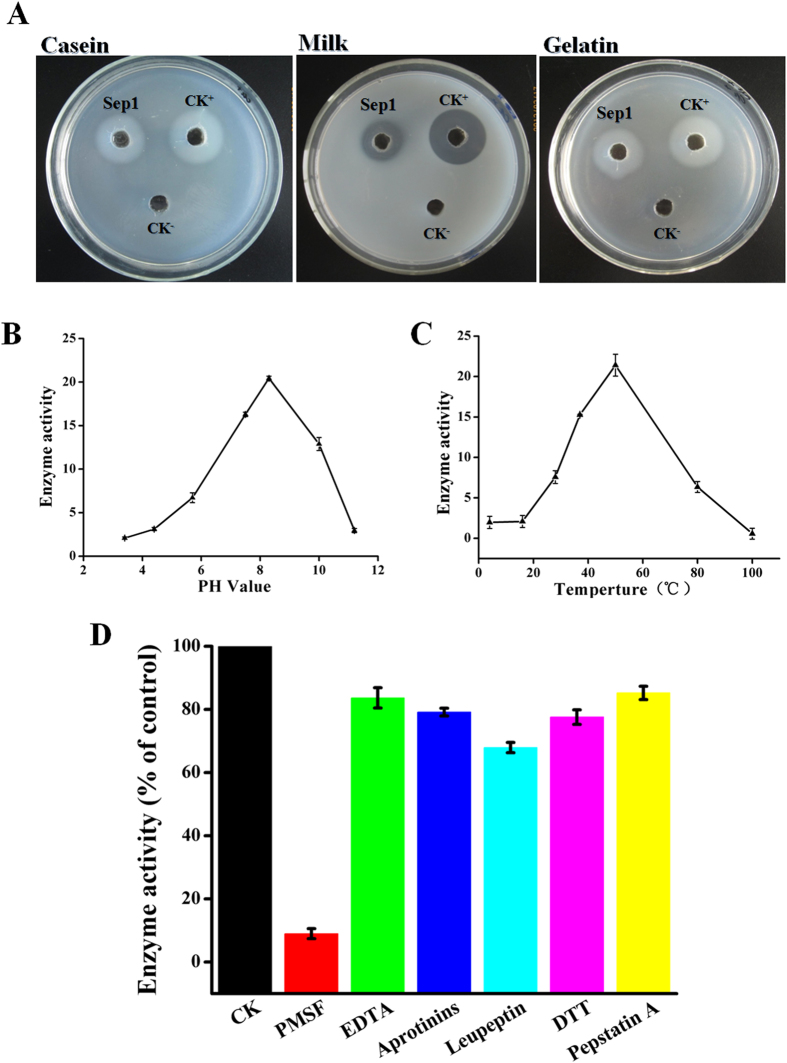
Sep1 protein exhibit serine protease activity. (**A**) The hydrolysis activity to different substrates of Sep1 by agar plate method. The effect of temperature (**B**), pH (**C**), and protease inhibitors (**D**) on Sep1 activity were conducted as the methods described before^49^. The concentration of protease inhibitors PMSF (1 mM), EDTA (1 mM), Aprotinins (2 μg/mL), Leupeptin (0.2 mM), DTT 5 (mM), and Pepstatin A (0.02 mM) were used according to the previously reports^49^. For the relative activity test, the casein as substrate and the assay system were adjusted for pH 8.0 and preincubation at 50 °C. One unit of enzyme was defined as the amount that caused the solubilization of 1 mg of protein per min under these assay conditions. The data are shown as means ± SD (n = 3).

The domain analysis showed that Sep1 is a potential subtilase, which has an Asp/His/Ser catalytic triad similar to that found in trypsin-like proteases^30^. Therefore, we used casein, milk, and gelatin as substrates to assess the hydrolytic activity of Sep1. The same concentration of trypsin was used as the positive control. The results showed that purified Sep1 could produce visible hydrolysis on casein, milk, and gelatin plates, similar to that of trypsin (Fig. 4A). These results indicate that the Sep1 protein has hydrolytic activity.

We then examined the effect of temperature, pH, and several protease inhibitors on Sep1 enzyme activity. The optimal catalytic conditions and enzyme stability were examined using casein as a substrate. The results of the pH studies indicate Sep1 possesses a broad pH tolerance and exhibited high stability of protease activity in a range of 6–11, with an optimum pH value of 8 (Fig. 4B). Sep1 also exhibited highly stable protease activity at a wide range of temperatures, from 30 to 80 °C, with an optimal temperature of 50 °C (Fig. 4C). To determine the nature of the purified enzyme, activity was measured in the presence of six reported protease inhibitors (Fig. 4D). When the protease inhibitors EDTA, aprotinin, leupeptin, DTT, and pepstatin A were added to the reaction system, the Sep1 protease activity significantly decreased, with a range from 67.91% to 85.24%. However, there was very low activity (8.95%) with the protease inhibitor PMSF (1.0 mM) (Fig. 4C). Considering that PMSF is a specific strong serine protease inhibitor^31^, and taking into account the conserved domain and hydrolysis activity information, we conclude that the Sep1 protein is a novel serine protease.

### The nematicidal activity of Sep1 is dependent on its serine protease activity

**Figure 5:**
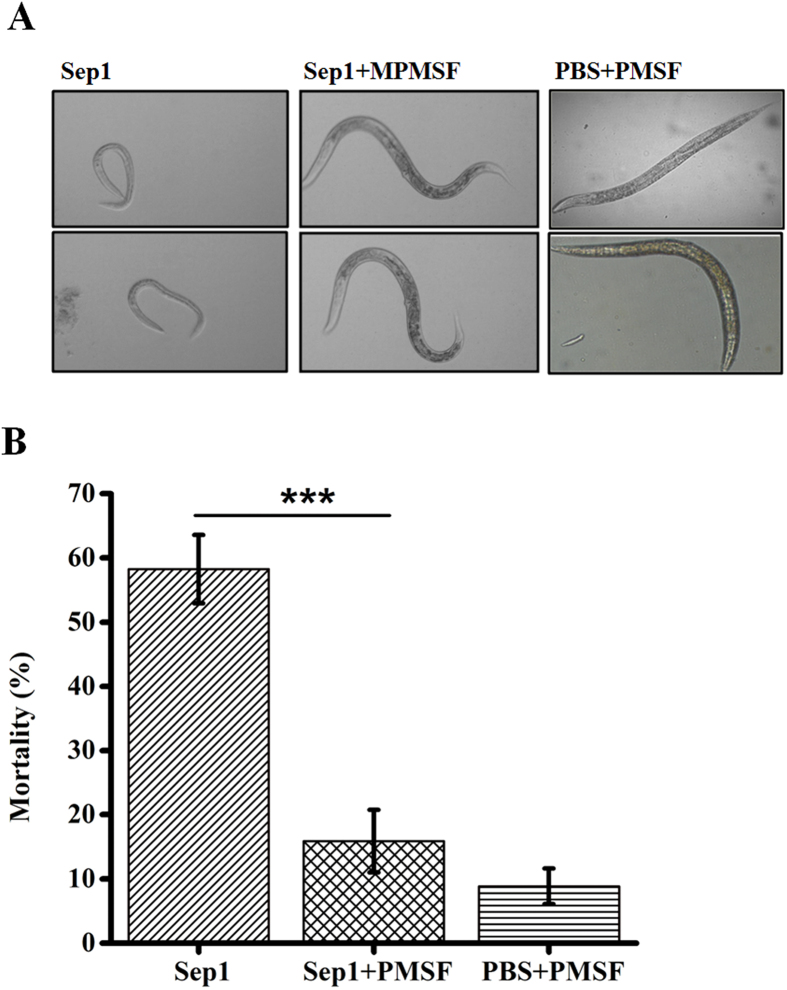
The nematocidal activity of Sep1 is depending on its serine protease activity. (**A**) The growth assays of 500 μg/ml Sep1 against *C. elegans* N2 L1 animals (20–30 for each well) with or without PMSF (1 mM) were conducted and observed under optical microscope. (**B**) Mortality assay of 500 μg/ml Sep1 against *M. incognita* J2 animals (50–60 for each well) with or without PMSF (1 mM) were conducted. The PBS buffer with 1 mM PMSF was used as control. The data are shown as means ± SD (n = 3).

We showed that the Sep1 protein has toxicity against *C. elegans* and *M. incognita.* Here, we confirmed that Sep1 exhibits serine protease activity. To evaluate whether the nematicidal activity of Sep1 is dependent on its enzyme activity, growth assays of Sep1 protein against *C. elegans* N2 L1 animals with or without the specific inhibitor PMSF were conducted. PBS buffer with 1 mM PMSF was used as a control. The results showed that animals challenged with 500 μg/ml Sep1 plus 1 mM PMSF were as healthy and vigorous as animals in the control group. However, the nematodes challenged with 500 μg/ml Sep1 were significantly smaller than the control group (Fig. 5A).

To evaluate whether the nematicidal activities of Sep1 against PPNs are dependent on its enzyme activity, a mortality assay of Sep1 protein against *M. incognita* J2 animals with or without the specific inhibitor PMSF was conducted. The results showed that 500 μg/ml Sep1 caused significant lethality (58.24%) in *M. incognita* J2 animals (Fig. 5B). However, when challenged with 500 μg/ml Sep1 plus 1 mM PMSF, the tested animals showed a significantly (*p* < 0.001) lower mortality (15.88%) compared with that of the 500 μg/ml Sep1-treated group, although the animals in the control group showed the lowest mortality of 8.85%. These results indicate that the nematicidal activity of Sep1 is dependent on its serine protease activity.

### Sep1 could destroy the intestinal integrity of *C. elegans* and *M. incognita*

Because Sep1 has similar conserved domains and enzymatic characteristics as the reported extracellular proteases from nematicidal bacteria^10^,^32^ and nematode-trapping fungi^26^,^27^,^28^, there is a possibility that Sep1 is an extracellular protease that can destroy the nematode physical barrier. To confirm this hypothesis, we then investigated whether the Sep1 protein degraded tissues from the nematodes *C. elegans* and *M. incognita*.

**Figure 6:**
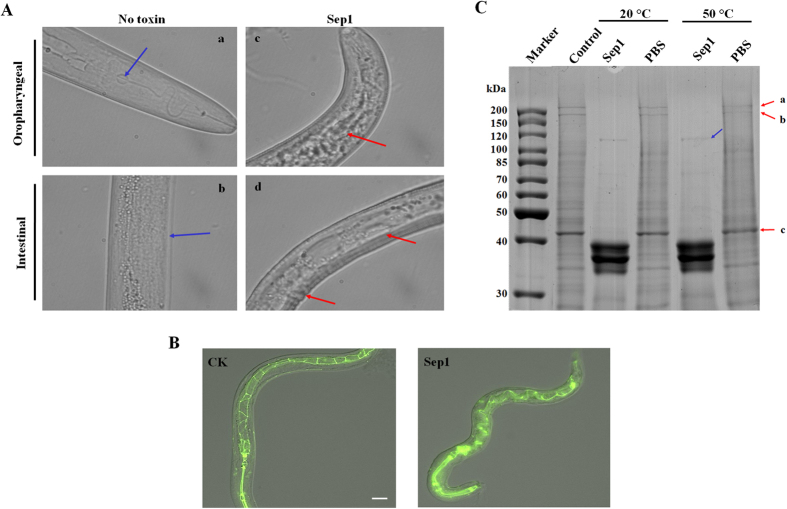
Sep1 destroy the tissue of *C. elegans* and *Meloidogyne incognita.* (**A**) The pathological characteristics of Sep1 target *M. incognita*. The tissues observed under a light microscope after feeding with Sep1. Pharyngeal grinder disrupt by Sep1(c) contrast with (a), intestinal cell destroyed by Sep1(d) contrast with (b), also indicate the cuticle destroyed (d) Nematodes J2 animals (30–40) were incubated with 500 μg/ml Sep1 protein for 48 h. The 1 μg/ml RES treated animals were used as controls. (**B**) The intestinal GFP labeled transgenic nematode FT63 (DLG::GFP) intestinal tissues observed under microscope after feeding with Sep1. Nematodes were incubated with 500 μg/ml Sep1 protein for 48 h. The ddH_2_0 treated nematodes were used as controls (CK). All nematodes are shown at the same magnification (scan bar 100 μm). **(C**) SDS-PAGE analysis showed Sep1 degraded the *C. elegans* tissue proteins. The red arrows point to the derivative proteins; the blue arrows indicate the products of some derivative proteins.

We first assessed the pathological characteristics of Sep1 against *M. incognita*. The J2 animals (50–60 for each well) of *M. incognita* were fed with 500 μg/ml Sep1 plus 1 μg/ml resorcinol (RES) for 48 h based on our previous work^33^, which stimulated the uptake of sep1. The 1 μg/ml RES solution was used as control. We then observed and compared the differences between the Sep1-treated group and the control group with an optical microscope (Fig. 6A). The results show that the pharyngeal tissues of the root-knot nematode became disorganized after Sep1 treatment and the esophageal balloon and stylet were severely damaged (red arrows shown in panel c). Additionally, the intestinal tissues also showed damage after Sep1 treatment (red arrows in panel d). Atrophy of the epidermal tissue of the intestine (red arrows in panel d) was also observed. These results demonstrate that the Sep1 protein could damage the *M. incognita* tissues.

We then assessed the effects of Sep1 on *C. elegans* tissue. We used a transgenic nematode FT63(DLG::GFP) with GFP labeling in the intestine and fed it 500 μg/ml Sep1 for 48 h based on our previous work^34^ and then examined the animals with fluorescence microscopy (Fig. 6B). In control animals, the worm intestine can be observed as segmented GFP fluorescence in the intestinal epithelial cells. For Sep1-treated nematodes, most of the regular ordered edges of the FT63 (DLG::GFP) intestine had been destroyed, resulting in disorganized or even absent GFP localization, while in other regions, such as the ventral or vesicular structures, GFP fluorescence was present, indicating a mislocalization phenotype. We concluded that Sep1 can destroy the intestinal integrity via epithelial cell protein damage from the above results.

We believed the Sep1 nematicidal activity was because the protein can destroy the intestine and cuticle of the nematode. To explore the role of Sep1 in targeting the nematode, we isolated total proteins from healthy *C. elegans* animals and incubated them with 500 μg/ml Sep1 *in vitro* at 20 °C (the nematodes normal growth temperature) and 50 °C (the optimal temperature for enzyme activity) for 1 h. Then, the profiles of the treated nematode proteins were detected by SDS-PAGE. We observed that when incubated with Sep1, a series of protein bands with molecular weights between 150–200 kDa and a major band at 45 kDa disappeared compared to the untreated samples (Fig. 6C). However, we noticed that there was an additional major band of 110 kDa that appeared in the untreated samples but not the Sep1-treated samples. It is likely that the nematode tissue proteins were degraded into small molecular weight fragments. These results indicate that Sep1 can degrade *C. elegans* structural proteins. We further investigated what types of proteins that can be degraded by Sep1 in *C. elegans*. We focused on the three degraded proteins (bands a, b, and c) in the gel in Fig. 6C. Then, we identified them with mass spectrometry. The best hits of bands a, b, and c were vitellogenin (vit-6), vitellogenin (vit-5) and actin (act-3), respectively. Notably, these three proteins are all reported as major structural proteins in the intestine or cuticle of *C. elegans* (http://www.wormbase.org). In sum, we conclude that Sep1 can target and degrade the intestinal proteins of free-living nematodes and PPNs, leading to the nematicidal activity against PPNs.

## Discussion

Plant-parasitic nematodes (PPNs) cause great harm to agricultural production worldwide^1^,^2^,^3^. At present, although many natural nematophagous fungi or bacteria have been used in the bio-control of PPNs, disadvantages such as culture technologies and host limitations still exist^35^. For example, nematicidal fungus are not easily produced and are inhibited by soil^8^, most nematicidal bacteria are only effective against larvae and eggs but cannot control adult PPNs^9^, and *Pasteuria penetrans* cannot be easily cultured aerobically^36^. Therefore, environmentally friendly, effective and affordable alternatives for PPN control are urgently needed. *B. firmus* shows excellent control of PPNs and has been produced as a commercial nematicide^21^. In the present study, we report the isolation a *B. firmus* strain DS-1 with nematicidal activity against *M. incognita* and soybean cyst nematode, which may open up new areas for studies on bacterial biocontrol agents for PPNs.

In the plant rhizosphere, many natural microorganisms interact with PPNs and plants. Some produce traps to capture and kill the worms externally, while others act as internal parasites and produce toxins and virulence factors to kill the nematodes from within^1^. The mechanisms of nematophagous fungi can be mainly categorized into four groups, including nematode-trapping fungi, endoparasitic fungi, egg-parasitic fungi, and toxin-producing fungi^1^,^8^. According to their mode of action against nematodes, nematophagous bacteria are classified into the following groups: obligate parasitic bacteria, such as *P. penetrans*^36^; opportunistic parasitic bacteria, such as *B. nematocida*; parasporal Cry protein-forming bacteria, such as *B. thuringiensis*^37^,^38^; rhizobacteria; endophytic bacteria; and symbiotic bacteria^1^,^9^. Additionally, some nematophagous bacteria act by interfering with nematode-plant recognition, competing for an ecological niche or nutrients, or inducing systemic resistance of plants to control the PPNs^9^,^39^. Because different lethal mechanisms could delay the development of resistance to the applied nematicidal microorganisms, different types of nematophagous microorganisms with diverse active mechanisms could be used as pairwise nematicides or for rotation sprays in the fields. Therefore, we need to obtain further information regarding the killing mechanisms of *Bacillus firmus*.

Understanding the mechanisms of the nematicidal *B. firmus* will aid in the development of novel bio-agents with increased efficacy for PPN control. Here, we sequenced the whole genome of *B. firmus* DS-1 and identified a novel nematicidal virulence factor, Sep1, which is a serine protease and has lethal activity against *C. elegans* and *M. incognita.* The nematicidal activity of Sep1 is mainly due to its enzymatic activity. The Sep1 protease targets and destroys the nematode cuticle or intestine tissues. Our discovery indicated that *B. firmus* DS-1 is likely to act as an internal parasite that produces toxins and virulence factors to kill the nematodes from within, which is similar to some other natural nematophagous bacteria^40^.

Many protease virulence factors from fungi or bacteria are reported to be toxic to nematodes. For example, *B. laterosporus* can secrete extracellular proteases to degrade the cuticle of nematodes^41^. The collagenase of *B. thuringiensis* could damage the intestine and is involved in pathogenesis of *C. elegans*^34^. *B. nematocida* can secrete neutral protease Bace16 and Bae16 to target nematode intestinal tissue^10^,^32^. For nematophagous fungi, infectious proteases participate in several steps of host infection^42^. Some extracellular proteases from nematode-trapping fungi could degrade the cuticle proteins of nematodes^26^,^27^,^28^. However, there is no information about proteases as nematicidal virulence factors in *B. firmus.* Here, we showed that the novel serine protease Sep1 is a nematicidal virulence factor in *B. firmus* DS-1. We also demonstrated that the targets of Sep1 in *C. elegans* are multiple intestinal and cuticle-associated proteins, showing that the mechanisms are similar to the infectious proteases from other nematophagous fungi or bacteria. Additionally, the sequence alignment using the NCBI database revealed the serine protease in more than 100 bacterial species. However, the functional verification is limited in different species. Our study is the first report of a serine protease as an important virulence factor against PPNs in *B. firmus*. It also provides a novel biological function for this type of protein as a reference for other pathogens. Our other significant discovery indicates that the protection of plants by direct application of this protease or by transformation of the toxin gene into plants may be feasible as a strategy for the control of nematodes. Our findings indicate that Sep1 could be used as a novel alternative or cooperative agent with others nematicides for durable PPNs control. Moreover, isolating new strains with high production of Sep1 protein should be promoted, as well as constructing *Bacillus licheniformis* or other commercial strains transformed with the *sep1* gene for PPN control; additionally, the *sep1* gene could be directly used in binary GM crops with Bt toxin^11^,^12^ for increased nematicidal activity.

It should be noted that the molecular mechanisms of nematicidal activity are very complex. Nematicidal activity is probably attributed to many factors, including extracellular proteases, toxic peptides, or other metabolic products. The comparative genomic analyses indeed revealed multiple potential adaptation and virulence factors in *B. firmus* DS-1 (Table 1). Moreover, the strain specifically expressing Sep1 did not reach the full virulence of the fermentation broth (Figs 1 and 3D). Therefore, the role of other putative virulence factors is unclear and needs further research. Additionally, the comparative genomic analyses also showed that *B. firmus* DS-1 contains abundant gene clusters of antibiotics and bacteriocins, such as bacterial carotenoids, which may also contribute to defense against first-line immunity-mediated ROS (reactive oxygen species) production and facilitate *B. firmus* colonization in the host nematode immune system. Moreover, the lasso peptide, which is a novel bacteriocin, could disrupt host microbiota homeostasis. It has been reported that some antibiotics and bacteriocins, such as thuringiensin or avermectin, have nematicidal activity against PPNs^43^,^44^. Therefore, it is necessary to determine whether these factors contribute to bacterial nematicidal activity in the future. Additionally, purified Sep1 was less virulent than the supernatant and, as a serine protease, was easily inhibited in bioassays. To promote a novel durable green treatment for PPNs, further studies are necessary to enhance Sep1 yields in *B. firmus* and also to test its environmental safety in free-living nematodes and humans, as its enzyme substrate are ubiquitous. If its biosafety is confirmed, developing Sep1 GM crops in combination with nematicidal Bt toxin could more easily solve agricultural problems and allow for scalable production.

## Materials and Methods

### Bacterial strains, plasmids, and culture conditions

The bacterial strains and plasmids used in this study are listed in Table S1. The *Escherichia coli* and *B. firmus* strains were grown on Luria-Bertani (LB) agar plates with appropriate antibiotics at 37 °C and 28 °C, respectively.

### Nematode strains and maintenance

The wild-type strain Bristol N2^45^ and the transgenic strain FT63 (*dlg::gfp*)^46^ of *Caenorhabditis elegans* were provided by the Caenorhabditis Genetics Center (http://www.cbs.umn.edu/CGC/). The transgenic nematode FT63, described as [dlg-1::GFP + rol-6(su1006)], expresses DLG-1::GFP in intestinal epithelial cell junctions and displays gut integrity. The *C. elegans* animals were cultured on NG plates at 20 °C and fed with *Escherichia coli* strain OP50 as food^45^. The L1 and L4 stage larvae were obtained as described^47^ for bioassays. *Meloidogyne incognita* was collected from the root knots of tomatoes. The soybean cyst nematode was provided by Dr. Deliang Peng (Institute of Plant Protection, Chinese Academy of Agricultural Sciences, Beijing, China).

### Bioinformatics prediction and sequence analysis

InterproScan analysis was performed on *Bacillus firmus* Ds-1 genome sequences with a conserved motif search, which included all the reported virulence factors, including Bace16^10^ of *Bacillus nematocida,* Bmp1 and all other proteases from *Bacillus thuringiensis*^25^, as well as the hemolysin superfamily proteins, peptidase_S8, peptidase_M6, peptidase_M7, and peptidase_M48 superfamily proteins.

Amino acid sequence alignments and phylogenetic trees were produced using the MEGA 4.1 software package. Primary and secondary protein structure predictions were carried out using the PHD program (http://www.predictprotein.org/). The protein sequences were compared to other proteins using BLAST. The conserved domains were predicted using the CDD database.

### Cloning, expression and purification of 14 putative virulence factors

Based on the genome sequence analysis, we cloned 14 putative virulence factor genes into the expression vector pET-28a. Briefly, the *sep1* gene as an example: the *sep1* gene was cloned by PCR from *B. firmus* DS-1 genomic DNA with a pair of specific primers, prt173-F and prt173-R. The *sep1* amplified fragment was then inserted into the *Eco*RI and *Hind*III sites of the vector pET-28a for expression with a His-tag. The recombinant plasmid was then transformed into *E. coli* BL21 (DE3), yielding the recombinant strain EMB2152. Finally, the Sep1 target protein was purified with His-Bind columns (Qiagen, Germany) according to the manufacturer’s instructions. The cloning, expression and purification of other putative virulence factors genes were performed as described for Sep1. The primers used here are listed in Table S2.

### RNA extraction and expression analyses

Total RNA was extracted from *B. firmus* DS-1 using TRIzol reagent (Invitrogen, California, USA) at different growth phases. Then, the cDNA was reverse transcribed with random primers using Superscript II reverse transcriptase (Invitrogen, California, USA) according to the manufacturer’s protocol. Analyses of the expression of *sep1* in strain DS-1 were performed using RT-PCR with the primers listed in Table S2. The 16s RNA gene was amplified as a reference gene for *sep1* in *B. firmus*.

### Nematode toxicity bioassay

The growth and lethality bioassays of Sep1 against *C. elegans* N2 were performed as previously described^29^ using Sep1 expressing *E. coli* BL21 with different diluted bacterial suspensions. The growth of the N2 L1 animals (20–30 for each dose) was observed under 100x magnification on a compound microscope. Then, the growth inhibition curve was generated after 3 days of treatment with Sep1. The inhibition percentage was the ratio of average worm size treated with *E. coli* BL21 expressing Sep1 relative to the average size of animals treated with control *E. coli*. The worm area was measured and calculated using ImageJ software^29^.

The purified Sep1 proteins were used for PPN bioassays. We conducted mortality tests of *M. incognita* and soybean cyst nematode according to a previous report^33^ to test the toxicity of the proteins against nematodes. Mortality was defined based on the observation of motility; a visibly moving nematode was marked as alive, and nematodes that failed to respond after several touches were marked as dead. The bioassays were repeated a minimum of three times to calculate the dose-dependent activity for the proteins.

### Protease assays

We followed the method with Bmp1 previously described^43^, with overnight precipitation instead, using the purified Sep1. Protein concentration was determined by the Bradford method using bovine serum albumin (BSA) as a standard. Protease activity was measured by adding 20 ug crude enzyme samples (diluted as needed) to 1 ml 10 mg/L protein substrate of casein solution (pH 7.0 or different pH value when needed) in a test tube followed by incubation at 37 °C for 10 min. Then, the reaction was stopped by adding 2 ml 1 M trichloroacetic acid, and the mixture was kept at 4 °C for 10 min. After overnight precipitation, the 1 ml supernatant was mixed with 5 ml 0.55 M sodium carbonate and 1 ml Folin-hydroxybenzene agent followed by incubation at 37 °C for another 10 min. Protease activity was measured with a spectrophotometer at 680 nm. A calibration curve using L-tyrosine as a standard was completed to measure production of L-tyrosine in the reaction. One unit (U) of protease activity was defined as the amount of enzyme that hydrolyzed the substrate and produced 1 ug tyrosine in 1 min under the assay conditions.

We used casein, milk, gelatin, and BSA as substrates to detect the protease activity of Sep1. Trypsin was used as a positive control. The effects of temperature, pH, metal ions, and protease inhibitors on the activity of the purified Sep1 were tested using a previously described method^25^. The temperature and pH ranges tested were 4–100 °C and pH 2–13, respectively. The protease inhibitors included phenylmethylsulfonyl fluoride (PMSF), ethylenediaminetetraacetic acid (EDTA), dichlorodiphenyltrichloroethane (DDT), aprotinin, leupeptin and pepstatin A. The assay system was adjusted to pH 8.0 and preincubated at 50 °C. One unit of enzyme was defined as the amount that caused the solubilization of 1 mg of protein per min under these assay conditions.

### Microscopic observation

Intestinal (DLG/AJM) GFP-labeled transgenic FT63 (*dlg::gfp*) worms were used to determine the integrity of the nematode intestinal epithelial cells. Under normal conditions, the FT63 (*dlg::gfp*) *C. elegans* intestine contained full clearly ordered “bamboo-like” GFP fluorescence under the fluorescence microscope. If the intestinal epithelial cell integrity was destroyed, the ordered edge lines would also be destroyed, resulting in disorganization or even disappearance of the GFP signal, but other areas, such as the bottom or vesicular structures, would continue to show GFP fluorescence (mislocalization).

### Mass spectroscopy analysis of protein bands excised from the SDS-PAGE gel

The target protein bands were excised from the SDS-PAGE gel. Then, the trypsin digestion peptides were examined using a matrix-assisted laser desorption ionization-time-of-flight (MALDI-TOF)/TOF-MS system (4700 Proteomics Analyzer, Applied Biosystems). Database searching of spectral data was carried out using the MASCOT search engine (www.matrixscience.com, Matrix Science).

### Data analysis

The data analysis was performed using SPSS (Statistical Package for the Social Sciences), version 13.0 software (SPSS, Chicago, IL, USA). LC_50_ values were calculated using PROBIT analysis^48^ and shown as the mean ± SD (n = 3). Statistical comparisons between two values were performed with a paired *t*-test, and significant differences were determined according to a threshold of *p < 0.05; **p < 0.01, and ***p < 0.001.

## Additional Information

**How to cite this article**: Geng, C. *et al.* A novel serine protease, Sep1, from *Bacillus firmus* DS-1 has nematicidal activity and degrades multiple intestinal-associated nematode proteins. *Sci. Rep.* **6**, 25012; doi: 10.1038/srep25012 (2016).

## Supplementary Material

Supplementary Information

## Figures and Tables

**Table 1:** List of potential nematocidal virulence factors in *B. firmus* DS-1 genome and their activity against nematode *C. elegans*.

Proteins	Annotation	Conserved domains	Reference
EWG12917	M6 family metalloprotease domain protein	Peptidase_M6	50
EWG12971	protease HtpX	Peptidase_M48	
EWG13047	M6 family metalloprotease domain protein	Peptidase_M6	
EWG13067	microbial serine proteinase	Peptidase_S8	40
EWG12508	peptidase M48 family protein	Peptidase_M48	
EWG12155	minor extracellular protease vpr	Peptidase_S8	
EWG11446	subtilase family protein	Peptidase_S8	
EWG10594	peptidase M48 family protein	Peptidase_M48	
EWG10706	hemolysinA	Hemolysin	25
EWG12667	bacillopeptidase F	Peptidase_M6	
EWG09329	hypothetical protein	Peptidase_M7	
EWG10090	serine protease, Sep1	Peptidase_S8	
EWG10233	hemolysin III family protein	Hemolysin III	
EWG13080	immune inhibitor A	Peptidase_M6	50
EWG11382	beta-lactamase family protein	Peptidase_M7	
EWG12667	bacillopeptidase F	Peptidase_S8	
EWG11793	acetyltransferase family protein	Peptidase_S8	
