# Supplementary material for: A novel serine protease, Sep1, from Bacillus firmus DS-1 has nematicidal activity and degrades multiple intestinal-associated nematode proteins
Source: Sci Rep. 2016 Apr 27;6:25012. doi: 10.1038/srep25012 (PMC4846997; doi:10.1038/srep25012)
Supplement: Supplementary Information [file srep25012-s1.doc]

**A novel serine protease, Sep1, from *Bacillus firmus* DS-1 has nematicidal activity and degrades multiple intestinal-associated nematode proteins**

Ce Geng^#^, Xiangtao Nie^#^, Zhichao Tang, Yuyang Zhang, Jian Lin, Sun Ming, Donghai Peng*****

State Key Laboratory of Agricultural Microbiology, College of Life Science and Technology, Huazhong Agricultural University, Wuhan 430070, Hubei, People’s Republic of China

*Corresponding author, Dr. Donghai Peng, Tel: 86-27-87283455; Fax: 86-27-87280670; E-mail: [donghaipeng@mail.hzau.edu.cn](mailto:donghaipeng@mail.hzau.edu.cn).

**^#^** These authors contributed equally to this work.

**Supplementary information:**

**Supplementary Figures:**

**Figure S1. The multiple sequence alignment of Sep1 with other selected nematocidal proteases.** The amino acid sequences were aligned with the ClustalW program. The species and GenBank accession numbers of other selected nematocidal proteases are shown. The “*”at the top indicate the conserved amino acid residues.


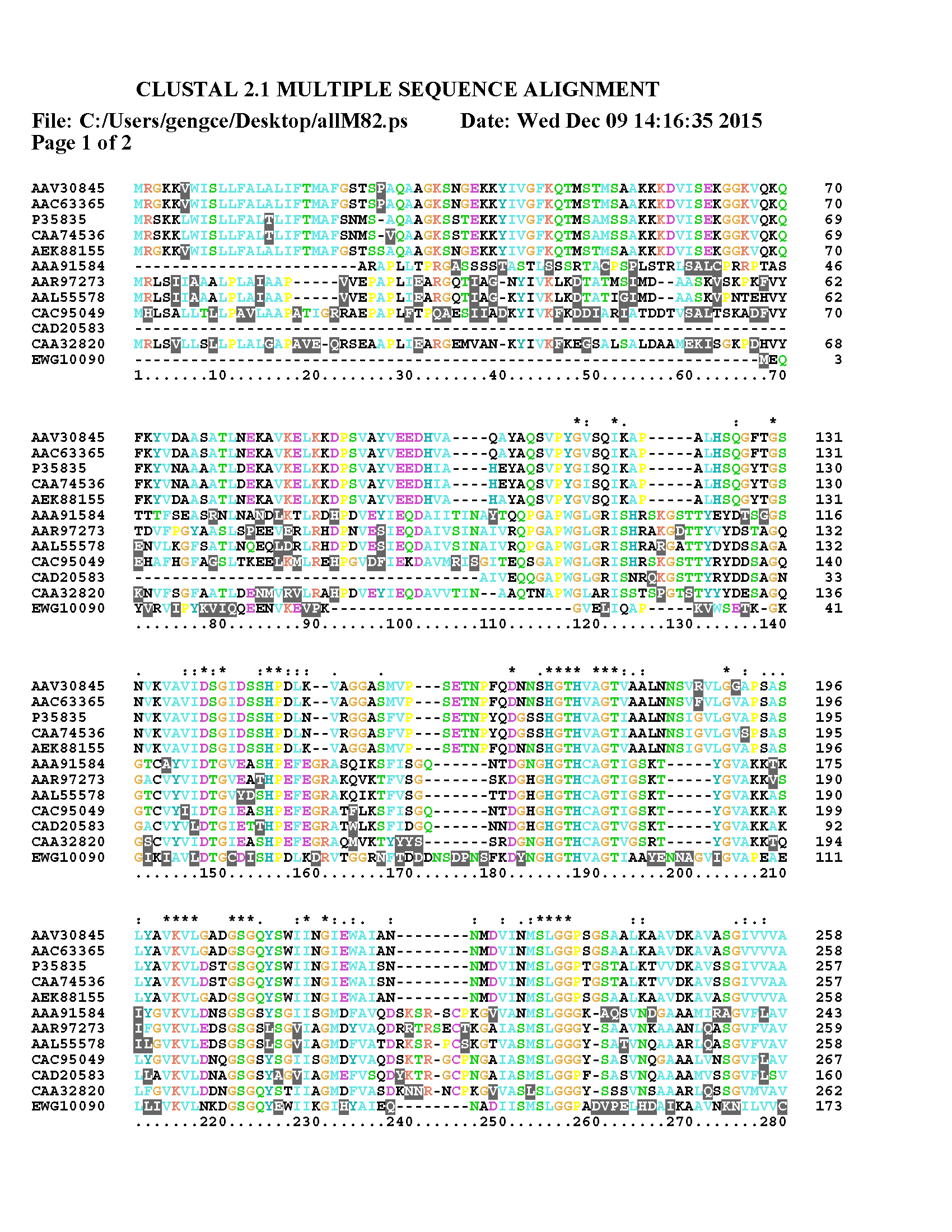


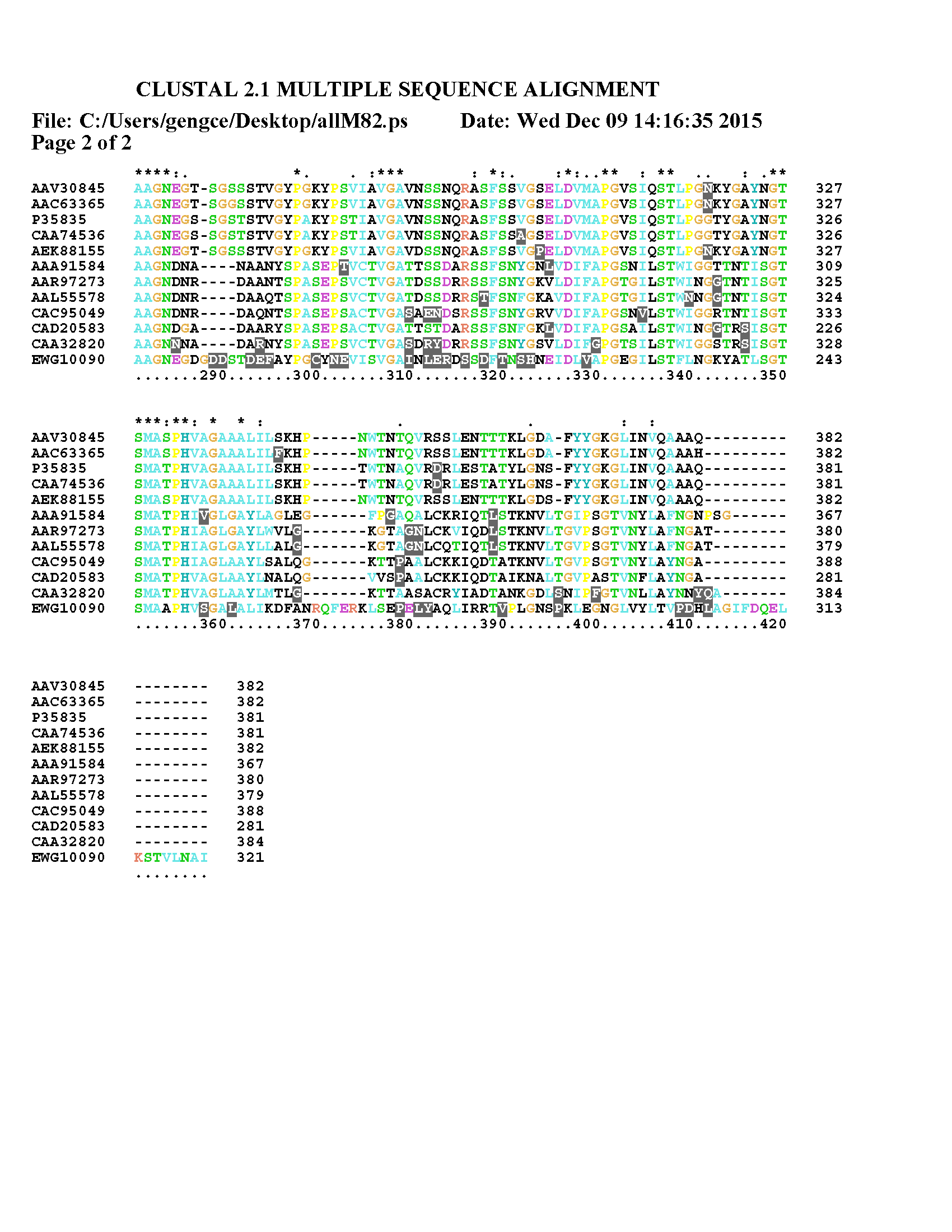


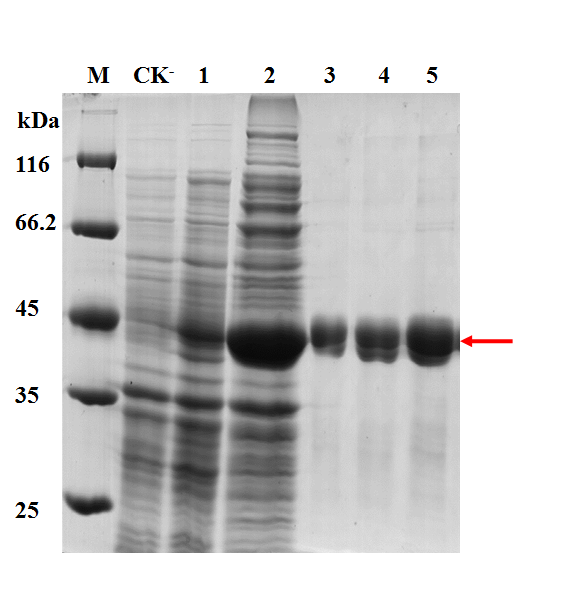


**Figure S2. The SDS-PAGE analysis of expression and purification of Sep1 protein in *E .coli*.**M, protein molecular maker; Ck-, pET28a/BL21(DE3) with 0.2 mmol/L IPTG induction; line 1, total proteins of pET28a-Sep1/BL21(DE3) with 0.2 mmol/L IPTG induction for 2 h; line 2, the proteins in soluble fraction of pET28a-Sep1/BL21(DE3) with 0.2 mmol/L IPTG induction for 2 h;lines 3-5, the purified Sep1 proteins from soluble fraction of pET28a-Sep1/BL21(DE3) with 0.2 mmol/L IPTG induction for 2 h.

**Figure S3. MALDI-TOF/TOF mass spectrophotometry results of peptide sequences in trypsin digestion proteins from SDS-PAGE gel bands.** The mass spectroscopy analysis of protein bands a, b, and c in Figure 6C.The result show the molecular weight of these three bands is 194466 Da, 187462 Da, and 42111 Da, and the sequence coverage is 45%, 64%, and 64%, respectively. The band a is the vitellogenin (vit-6) (A); band b is the vitellogenin (vit-5) (B); and band c is the actin (act-3) (C).The red font indicates the detected matched peptides in the positive hits amino acids sequence. Figure S3A:


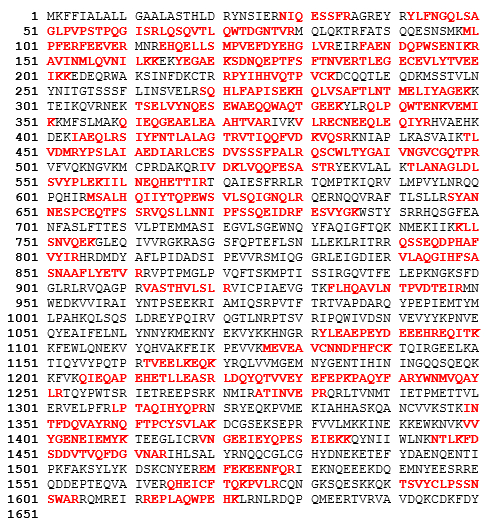


**Figure S3B:**


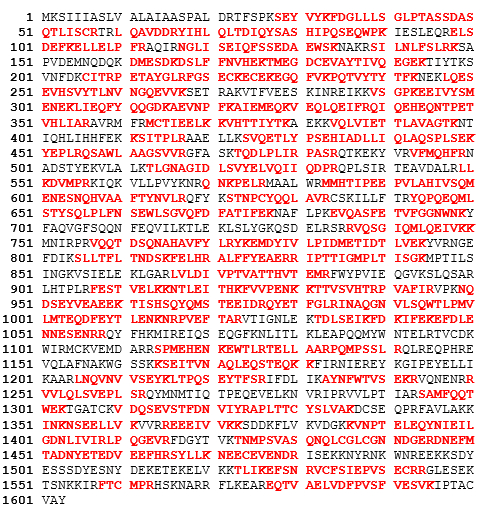


**Figure S3C:**


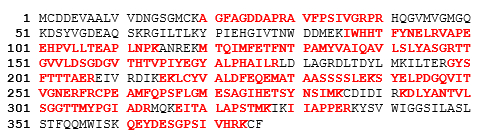


**Supplementary Tables**

**Table S1. Bacterial strains and plasmids used in this study.**

| **Strains and plasmids** | **Characteristics** | **Origin** |
| --- | --- | --- |
| ***Escherichia coli* strains** | | |
| OP50 | OP50 *ura* | Lab collection |
| BL21(DE3) | F^-^*ompThsdS*_B_(r_B_^-^m_B_^-^) *gal dcm* (*DE3*) | Lab collection |
| EMB2160 | Derivative of BL21 containing pET28a plasmid for blank vector expression | This work |
| EMB2141 | Derivative of BL21 containingpBMB2121 for producing EWG12917 protein. Kan^r^ | This work |
| EMB2142 | Derivative of BL21 containingpBMB2122 for producing EWG12971 protein. Kan^r^ | This work |
| EMB2143 | Derivative of BL21 containingpBMB2123 for producing EWG13047 protein. Kan^r^ | This work |
| EMB2144 | Derivative of BL21 containingpBMB2124 for producing EWG13067 protein. Kan^r^ | This work |
| EMB2145 | Derivative of BL21 containingpBMB2125 for producing EWG12508 protein. Kan^r^ | This work |
| EMB2146 | Derivative of BL21 containingpBMB2126 for producing EWG12155 protein. Kan^r^ | This work |
| EMB2147 | Derivative of BL21 containingpBMB2127 for producing EWG11446 protein. Kan^r^ | This work |
| EMB2148 | Derivative of BL21 containingpBMB2128 for producing EWG10594 protein. Kan^r^ | This work |
| EMB2150 | Derivative of BL21 containing pBMB2130 for producing EWG12667 protein. Kan^r^ | This work |
| EMB2151 | Derivative of BL21 containing pBMB2131for producing EWG09329 protein. Kan^r^ | This work |
| EMB2152 | Derivative of BL21 containing pBMB2132 for producingSep1protein. Kan^r^ | This work |
| EMB2153 | Derivative of BL21 containing pBMB2133 for producing EWG10233 protein. Kan^r^ | This work |
| EMB2154 | Derivative of BL21 containing pBMB2134 for producing EWG13080 protein. Kan^r^ | This work |
|  |  |  |
| **Plasmids** |  |  |
| pET28-a | A expression vector, ori*E. coil,* Kan^r^, 5.4 kb. | Promega Corp. |
| pBMB2121 | Derivative of pET28a containing condingORF gene for producing EWG12917 protein. Kan^r^ | This work |
| pBMB2122 | Derivative of pET28a containing condingORF gene for EWG12971 protein expression. Kan^r^ | This work |
| pBMB2123 | Derivative of pET28a containing condingORF gene for EWG13047 protein expression. Kan^r^ | This work |
| pBMB2124 | Derivative of pET28a containing condingORF gene for EWG13067 protein expression. Kan^r^ | This work |
| pBMB2125 | Derivative of pET28a containing condingORF gene for EWG12508 protein expression. Kan^r^ | This work |
| pBMB2126 | Derivative of pET28a containing condingORF gene for EWG12155 protein expression. Kan^r^ | This work |
| pBMB2127 | Derivative of pET28a containing condingORF gene for EWG11446 protein expression. Kan^r^ | This work |
| pBMB2128 | Derivative of pET28a containing condingORF gene for EWG10594 protein expression. Kan^r^ | This work |
| pBMB2130 | Derivative of pET28a containing condingORF gene for EWG12667 protein expression. Kan^r^ | This work |
| pBMB2131 | Derivative of pET28a containing condingORF gene for EWG09329 protein expression. Kan^r^ | This work |
| pBMB2132 | Derivative of pET28a containing condingORF gene for Sep1 protein expression. Kan^r^ | This work |
| pBMB2133 | Derivative of pET28a containing condingORF gene for EWG10233 protein expression. Kan^r^ | This work |
| pBMB2134 | Derivative of pET28a containing condingORF gene for EWG13080 protein expression. Kan^r^ | This work |

**Table S2. Primers used in this study.**

| **Name** | **Sequence (5’-3’)** | **Used in plasmid** | **Function** |
| --- | --- | --- | --- |
| prt180-F | CGCGTCGACATGACCATTTCTTCAGCGG  CCGCTCGAGTTATTTCCCCGCACCCGG | pBMB2121 | EWG12917 expression |
| prt180-R |
| prt241-F | CGCGTCGACATGGGTAAACGTATTTTTTAC  CCGCTCGAGTTACTTCGCACTCAAACGTC | pBMB2122 | EWG12971 expression |
| prt241-R |
| prt322-F | CGCGTCGACATGCCTGGACAAGTCCCG  CCGCTCGAGTTATTTTGTACTGCTGTTCG | pBMB2123 | EWG13047 expression |
| prt322-R |
| prt344-F | CGCGTCGACATGAAGAAAAAAAGGGC  CCGCTCGAGTTATTTTACGCGAGGG | pBMB2124 | EWG13067 expression |
| prt344-R |
| prt259-F | CGCGTCGACATGGCGAGGAAATTAGGC  CCGCTCGAGTCAGTCTGCTGTCTGC | pBMB2125 | EWG12508 expression |
| prt259-R |
| prt139-F | CGCGTCGACATGAAAAAATTCTTATTG  CCGCTCGAGTCACTTGCTTAGTAACC | pBMB2126 | EWG12155 expression |
| prt139-R |
| prt7-F | CGCGTCGACATGAGGAGGAAGGGAATG  CCGCTCGAGCTATTGCAAGTTAACTTTC | pBMB2127 | EWG11446 expression |
| prt7-R |
| prt157-F | CGCGTCGACATGGAAGCTCACTCGCTG  CCGCTCGAGTTATGGCTGCAGGATTTCC | pBMB2128 | EWG10594 expression |
| prt157-R |
| prt95-F | CGCGTCGACATGAAAAAGATGTTCACT  CCGCTCGAGTTAGTAGACATTGTAAAC | pBMB2130 | EWG12667 expression |
| prt95-R |
| prt15-F | CGCGTCGACTTGAAAAGAAAAAGAAAATC  CCGCTCGAGTTACTTCTTTTTAGTGCTG | pBMB2131 | EWG09329 expression |
| prt15-R |
| prt173-F | GGAATTCATGGAGCAATATGTTCGTGT  CCCAAGCTTTCATATGGCATTAAGAACAG | pBMB2132 | Sep1/  EWG10090 expression |
| prt173-R |
| prt8-F | CGCGGATCCATGAATTCGTACATCCGGGAG  CCGCTCGAGCTATATAACATACATAAAAAC | pBMB2133 | EWG10233 expression |
| prt8-R |
| prt357-F | CCGGAATTCATGGCAGCTGCACTATTTGC  CCGCTCGAGTTACTTGTGAATCCATACGGC | pBMB2134 | EWG13080 expression |
| prt357-R |
| F-sep1-rt | GCAATATGTTCGTGTAAT |  | To analysis the transcription of *sep1* mRNA in DS-1 with a 200 bp product. |
| R-sep1-rt | TGTTATCATCGTCTGTAA |  |
| F-16S | AGACACGGCCCAGACTCCTAC |  | To analysis the transcription of 16S rRNA, this is a reference gene in *Bacillus firmus*. |
| R-16S | GACCCGAAAGCCTTCATCACT |  |

^a^The nucleotide sequences with underline indicated the restriction enzyme sites used in direct cloning.
